# CRISPR/Cas9‐mediated efficient targeted mutagenesis in grape in the first generation

**DOI:** 10.1111/pbi.12832

**Published:** 2017-11-10

**Authors:** Xianhang Wang, Mingxing Tu, Dejun Wang, Jianwei Liu, Yajuan Li, Zhi Li, Yuejin Wang, Xiping Wang

**Affiliations:** ^1^ State Key Laboratory of Crop Stress Biology in Arid Areas College of Horticulture Northwest A&F University Yangling Shaanxi China; ^2^ Key Laboratory of Horticultural Plant Biology and Germplasm Innovation in Northwest China Ministry of Agriculture Northwest A&F University Yangling Shaanxi China

**Keywords:** CRISPR/Cas9, genome editing, grape transformation, molecular breeding, *Vitis vinifera*

## Abstract

The clustered regularly interspaced short palindromic repeats‐associated protein 9 (CRISPR/Cas9) system is a powerful tool for editing plant genomes. Efficient genome editing of grape (*Vitis vinifera*) suspension cells using the type II CRISPR/Cas9 system has been demonstrated; however, it has not been established whether this system can be applied to get biallelic mutations in the first generation of grape. In this current study, we designed four guide RNAs for the *VvWRKY52* transcription factor gene for using with the CRISPR/Cas9 system, and obtained transgenic plants via Agrobacterium‐mediated transformation, using somatic embryos of the Thompson Seedless cultivar. Analysis of the first‐generation transgenic plants verified 22 mutant plants of the 72 T‐DNA‐inserted plants. Of these, 15 lines carried biallelic mutations and seven were heterozygous. A range of RNA‐guided editing events, including large deletions, were found in the mutant plants, while smaller deletions comprised the majority of the detected mutations. Sequencing of potential off‐target sites for all four targets revealed no off‐target events. In addition, knockout of *VvWRKY52* in grape increased the resistance to *Botrytis cinerea*. We conclude that the CRISPR/Cas9 system allows precise genome editing in the first generation of grape and represents a useful tool for gene functional analysis and grape molecular breeding.

## Introduction

Grape (*Vitis vinifera* L.) is a widely cultivated perennial fruit crop that has great economic value as it is a source of many products, including wine, jam, juice and jelly, grape seed extracts, raisins, vinegar and grape seed oil (Tu *et al*., 2016). However, the yield and berry quality of grape is limited by a range of biotic and abiotic stresses (Li, 2015). There is therefore considerable interest in understanding the molecular mechanisms that grape has evolved to resist such stresses. Most functional studies of grape resistance genes have used overexpression analysis (Dai *et al*., 2015); however, as this approach does not always accurately reflect normal gene function (Lloyd, 2003), gene silencing methods are now also being widely employed. To date, RNAi, VIGS (virus‐induced gene silencing) and gene editing have been the most commonly used means for gene silencing (Pandey *et al*., 2016; Shan *et al*., 2013; Wei *et al*., 2017).

Gene editing directly produces mutations in the genome sequence, which has advantages over RNAi and VIGS, and the CRISPR/Cas9 system is a new technology that provides a relatively straightforward means of plant genome targeting (Xing *et al*., 2014). This system, adapted from *Streptococcus pyogenes*, has emerged as an effective tool for gene functional analysis and molecular breeding in plant (Barabaschi *et al*., 2016), and operates through guide RNA sequences that contain specific targets designed according to the target genome sequence. The other core component of the CRISPR/Cas9 system is the Cas9 protein, which cleaves the specific location within the gene target. Compared with other genome editing systems, such as the Zinc Finger Nucleases and TALENs (transcription activator‐like effector nucleases), this system is relatively easy to deploy (Baltes and Voytas, 2015; Voytas and Gao, 2014) and has a wider range of potential applications prospect. Indeed, many CRISPR/Cas9 systems have been adapted for plant genome editing (Ma *et al*., 2016). An important factor in the efficiency of this system is high expression of the Cas9 protein and effective targets, although designing two or more targets for one gene can improve the probability of obtaining homozygous mutations in the first generation (Fan *et al*., 2015). This is particularly important for woody plants, such as grape or apple (*Malus domestica*), which have long generation times. To date, there have been reports describing the use of CRISPR/Cas9 with the woody plants, such as orange (*Citrus reticulate*) (Jia and Wang, 2014), poplar (*Populus tomentosa Carr*.) (Fan *et al*., 2015; Zhou *et al*., 2015), apple (Malnoy *et al*., 2016; Nishitani *et al*., 2016) and grape (Malnoy *et al*., 2016; Ren *et al*., 2016; Wang *et al*., 2016b); however, it has also been used in a broad range of nonwoody plants, including *Arabidopsis thaliana* (Gao *et al*., 2016; Hahn *et al*., 2017; Jiang *et al*., 2013, 2014; Li *et al*., 2013, 2014; Liu *et al*., 2015; Mao *et al*., 2016; Peterson *et al*., 2016; Pyott *et al*., 2016; Shen *et al*., 2017; Tsutsui and Higashiyama, 2017; Yan *et al*., 2015; Zhang *et al*., 2016b), rice (*Oryza sativa*) (Li *et al*., 2017b; Lu and Zhu, 2017; Mikami *et al*., 2015; Sun *et al*., 2016; Zhang *et al*., 2014; Zhou *et al*., 2014a), tomato (*Solanum lycopersicum*) (Ito *et al*., 2015; Pan *et al*., 2016; Ueta *et al*., 2017; Van Eck *et al*., 2015), potato (*Solanum tuberosum*) (Andersson *et al*., 2017), cotton (*Gossypium hirsutum* L.) (Li *et al*., 2017a), soya bean (*Glycine max*) (Cai *et al*., 2015; Du *et al*., 2016; Jacobs *et al*., 2015; Li *et al*., 2015; Sun *et al*., 2015), maize (*Zea mays*) (Char *et al*., 2017; Feng *et al*., 2016; Qi *et al*., 2016; Shi *et al*., 2017; Svitashev *et al*., 2015, 2016; Zhu *et al*., 2016), sorghum (*Sorghum bicolor*) (Jiang *et al*., 2013), wheat (*Triticum aestivum*) (Gil‐Humanes *et al*., 2017; Liang *et al*., 2017; Shan *et al*., 2014; Upadhyay *et al*., 2013; Zhang *et al*., 2016a) and mosses (*Physcomitrella patens*) (Collonnier *et al*., 2017; Lopez‐Obando *et al*., 2016).

As the CRISPR/Cas9 technology has proven so effective, an increasing number of gene functional studies are based on this system. In *A. thaliana*, for example, the *cbf1 cbf3 and cbf1 cbf2 cbf3* (*cbfs*) mutants were generated by mutating *CBF1* and *CBF1/CBF2* in a *cbf3* T‐DNA insertion mutant via CRISPR/Cas9‐mediated system, and the resulting lines used to study responses to chilling and freezing stresses (Jia *et al*., 2016). An example from rice is the knockout of the ERF transcription factor gene, *OsERF922*, via CRISPR/Cas9, which gave rise to improved resistance to the rice blast fungus (Wang *et al*., 2016a). In maize, a *ARGOS8* mutant generated by the CRISPR‐Cas9 system showed increased resistance to drought stress (Shi *et al*., 2017), and in tomato, the mutations of *RIN* gene induced by CRISPR/Cas9 affected fruit ripening of T0 plants, while the mutations were shown to be stably inherited in the T1 generation (Ito *et al*., 2015).

In grape, five CRISPR/Cas9 target sites were identified and characterized (Wang *et al*., 2016b), and other reports have shown that this genome editing approach can be used with grape suspension cells or protoplast (Malnoy *et al*., 2016; Ren *et al*., 2016). However, little is known regarding the efficiency of CRISPR/Cas9‐mediated targeted mutagenesis in the first generation of grape mutants. For example, it is important for gene functional analysis and molecular breeding strategies to establish whether homologous mutations can be efficiently generated. In this study, we chose to target a gene (*VvWRKY52*) from the grape WRKY transcription factor family, which has been shown to play roles in biotic stress responses (Wang *et al*., 2017). Four specific targets were designed in the first exon region of *VvWRKY52*, and 22 independent mutant strains were generated, of which 15 were homozygous. We analysed the variation caused by four different target sites and confirmed that the technology can be efficiently used to generate homozygous mutations in grape in the first generation. In addition, we also demonstrated that the CRISPR/Cas9 system can be used for precise genome editing in the first generation of grape.

## Results

### Target selection and vector construction

**Figure 1:**
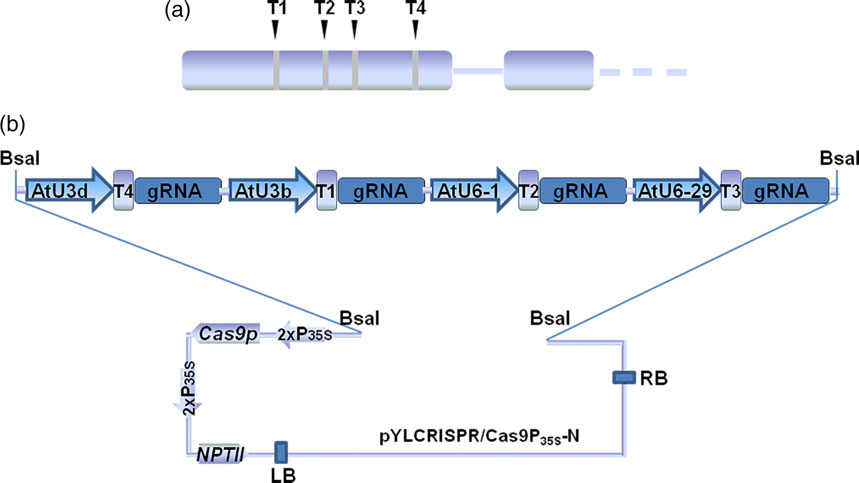
Target site selection in the *VvWRKY52* gene and the construction of Cas9/sgRNA. (a) Four sgRNAs (T1, T2, T3 and T4) were selected corresponding to sites in the first exon of *VvWRKY52*. (b) Schematic diagram of the protocol for constructing the four expression cassettes in the binary vector. Four *Arabidopsis thaliana* promoters, AtU3d, AtU3b, AtU6‐1 and AtU6‐29, were used to drive the four targets, T4, T1, T2 and T3, respectively. The four sgRNA expression cassettes were inserted into the binary vector with *Bsa*I.

The *VvWRKY52* gene is located on chromosome 16 in grape reference genome (Figure S1), and no other copy of the *VvWRKY52* was found in the Grape Genome Database (12×; http://www.genoscope.cns.fr). In addition, the whole genome of the *V. vinifera* Thompson Seedless cultivar was sequenced in 2014 (Di Genova *et al*., 2014), and the copy number variation (CNV) regions were identified between the grape reference genome (PN40024) and Thompson Seedless (Cardone *et al*., 2016). However, no CNV was found at the region of *VvWRKY52*. This indicated that *VvWRKY52* only has a single copy in the genome of Thompson Seedless. We also found two kinds of sequences at the target region of *VvWRKY52*, which are shown in Figure S1, allele I and allele II. According to the sequence results, 102 of 203 clones were allele I and others were allele II. This indicated that the sequences of these two sites are equal in the genome. We also analysed the mutant information at target 1 and target 4 in the mutant lines (Tables S3 and S4), and the results showed that only one kind of mutant type or wild‐type (WT) type was found in each allele of the mutant lines. Given that the mutation caused by this CRISPR/Cas9 system is random, each allele should have more than one mutant type at every target site in most mutant lines if the *VvWRKY52* has other copy in the grape genome. Therefore, we think that *VvWRKY52* only has a single copy in the genome of Thompson Seedless. The sequence of *VvWRKY52* gene was amplified with gene‐specific primers from genomic DNA of the *V. vinifera* Thompson Seedless cultivar, and used for target selection. The four targets were designed using the online tools CRISPR‐P (http://cbi.hzau.edu.cn/crispr/) and CRISPR RGEN (http://www.rgenome.net/), based on their GC content and putative off‐target sites (Table 1). Then, we confirmed that no SNPs were detected at four target sites compared with the Thompson Seedless genome sequence. All selected targets were located in the first exon and their relative position is shown in Figure 1a. Four *A. thaliana* promoter sequences, AtU3d, AtU3b, AtU6‐1 and AtU6‐29, were used to drive expression of the T4, T1, T2 and T3 targets, respectively (Figure 1b). The expression cassettes including the four targets were inserted into the binary vector pYLCRISPR/Cas9P35S‐N (Ma *et al*., 2015) using *Bsa*I (Figure 1b).

**Table 1:** Summary of the four selected targets

Target	Sequence	GC%	Number of putative off‐target sites
1	ACATGACGCCCGTGAATCCTTGG	55	3
2	GCTGAGGTGTAGCGGCCCAGTGG	70	2
3	CACAGGCCGCCGCAGCAGGCGG	80	6
4	AGTCTCCACGCTCGCTCAGTGG	65	2

### Grape transformation and identification of transgenic mutant lines

**Figure 2:**
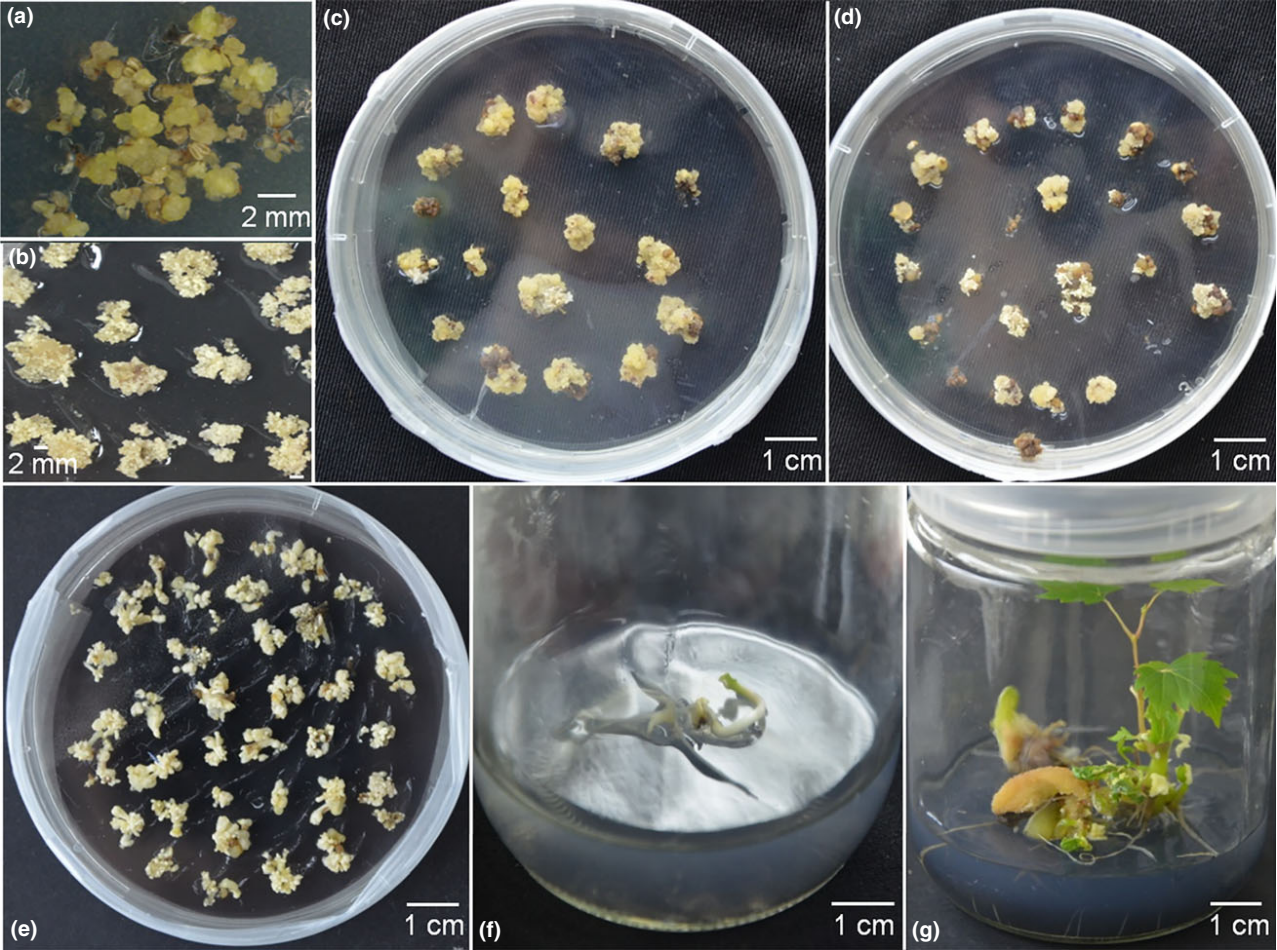
Induction of proembryonal masses (PEM) and grape transformation. (a) Callus induction from flower buds. (b) PEM induction from callus on X6 medium. (c) Kanamycin‐resistant embryogenic callus (EC) on X6 medium with 200 mg/L carbenicillin, 200 mg/L cefotaxime and 75 mg/L kanamycin (X6CCK75). (d) The formation of PEM from kanamycin‐resistant embryogenic callus on X6CCK75 medium. (e) The formation of somatic embryos from PEM X6CCK75 medium. (f) and (g) Somatic embryo germination and plantlet formation on MS1B medium.

**Figure 3:**
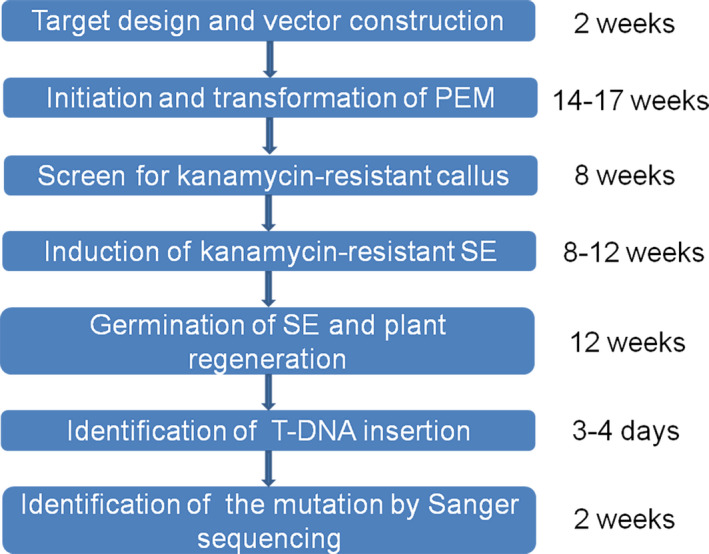
A flow chart of CRISPR‐based mutagenesis in grape using Agrobacterium‐mediated transformation method. A minimum of 12 months is required from test period.

To generate the proembryonal masses (PEM) used for grape transformation, flower buds of Thompson Seedless were selected for embryo callus induction (Figure 2a) (Dhekney *et al*., 2012). The embryo callus was then transferred to X6 medium for 1–3 weeks and maintained in the dark at 26 °C. After 3–5 weeks, the PEM developed on the X6 medium (Figure 2b). *Agrobacterium tumefaciens* strain EHA105, containing the CRISPR/Cas9 vector, was used to transform the PEM as previously described (Dhekney *et al*., 2012). After co‐culturing, the PEM was transferred to solid DM medium for callus induction, and the re‐induced callus was then transferred to X6 medium with 200 mg/L carbenicillin, 200 mg/L cefotaxime and 75 mg/L kanamycin (X6CCK75) (Figure 2c). The kanamycin‐resistant PEM (Figure 2d) was obtained after maintaining the callus in the dark at 26 °C on X6CCK75 medium for 4–6 months, and the somatic embryos (SE) (Figure 2e) developed from the PEM were selected for transgenic plant recovery (Figure 2f,g). The entire experiment cycle was approximately 12 months, dating from the target design to the identification of mutant transgenic lines (Figure 3).

**Figure 4:**
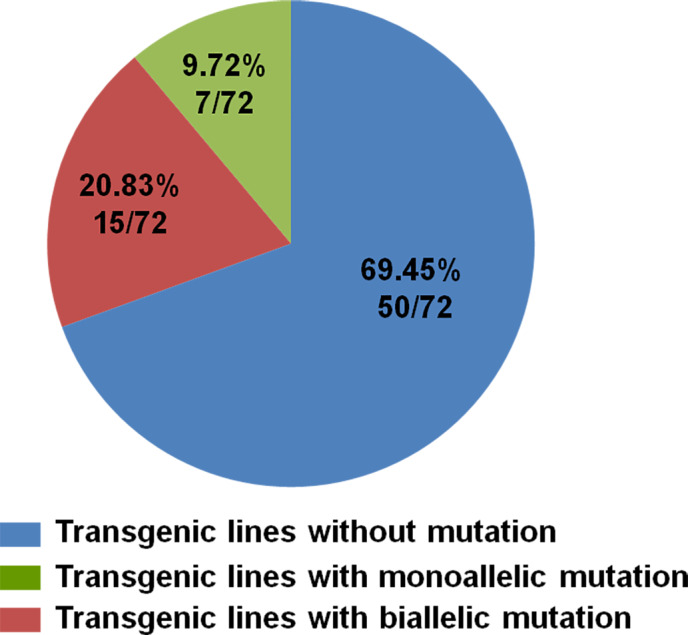
The efficiency of the CRISPR/Cas9 system in grape. Seventy‐two lines were identified with the T‐DNA insertion, 22 (15 + 7) of which mutations and, of these, 15 of these had mutations that were bialleles.

Genomic DNA was extracted from leaves of transgenic lines and WT plants, and PCR was performed to confirm presence of the transgene using vector‐specific primers (*NPTII*‐F: 5′‐AGAGGCTATTCGGCTATGACTG‐3′; *NPTII*‐R: 5′‐CAAGCTCTTCAGCAATATCACG‐3′). All 72 putative transgenic plants tested positive in this regard (Figure S2). In order to detect the mutations in the transgenic plants, the putative edited area of *VvWRKY52* was amplified by gene‐specific primers (*VvWRKY52*‐Target‐F; *VvWRKY52*‐Target‐R) from all the transgenic lines and WT plants (Figure S3), and the PCR products were sequenced. Of the 72 transgenic lines, 22 (31%) contained mutations (Figure 4). The PCR products from each of these 22 mutant lines were inserted in the pClone007 Simple Vector (TSINGKE, Xi'an, China) and 5–24 single clones from each line were sequenced. According to the sequence results, two kinds of sequences at target region of *VvWRKY52* are shown in Figure S1. In view of *VvWRKY52* as a single copy gene, we think these sequences come from two different alleles, one inherited from the mother and another from the father. The information was used to identify the biallelic mutant lines.

**Figure 5:**
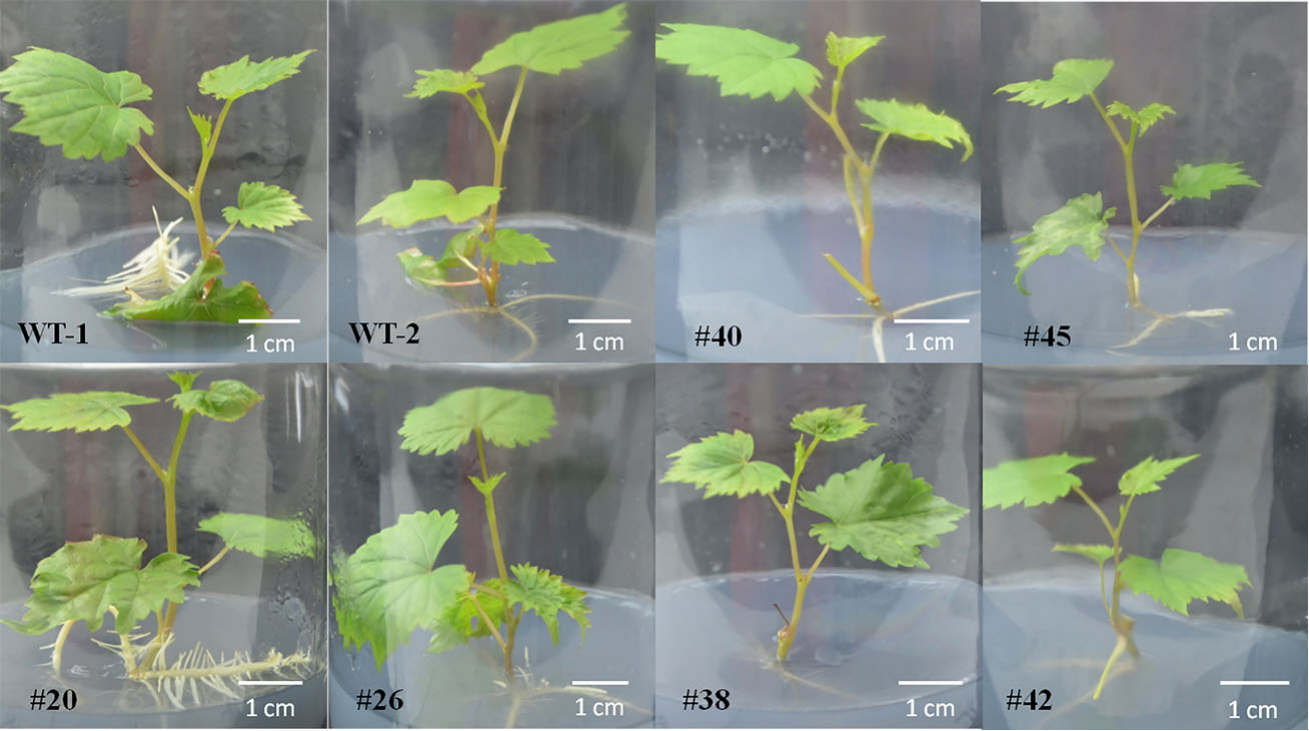
The phenotype of wild type (WT) and transgenic lines (#20, #26, #40, #45, #38, #42). Lines #20 and #26 had the T‐DNA insertion but no mutations in the *VvWRKY52* sequence. Lines #40 and #45 contained single allele mutations, and lines #38 and #42 contained biallelic mutations.

Fifteen of 22 mutant lines carried biallelic mutations, while the remaining had only mutations on one allele (Figures 4 and 7a). The morphologies of the different transgenic lines and WT plants are shown in Figure 5. No significant differences were found between the phenotypes of the WT and transgenic lines #20, #26, #40, #45, #38 and #42.

### Identification of CRISPR/Cas9‐induced mutations in *VvWRKY52*

**Figure 6:**
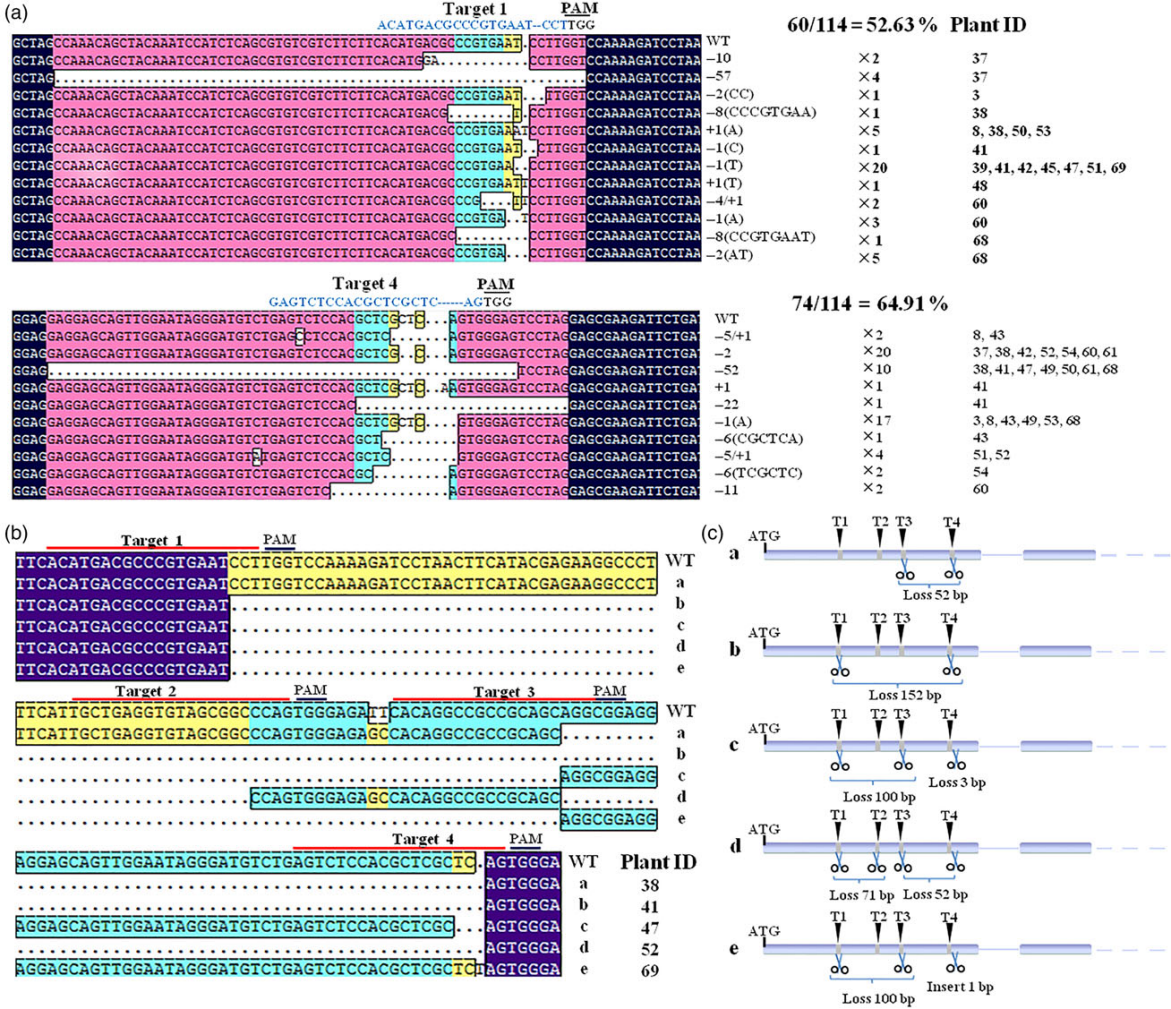
Different types of mutations detected in the transgenic grape after CRISPR/Cas9‐mediated gene editing. (a) The insertion and deletion mutations in target 1 of the T0 transgenic plants. The numbers of clones detected with this mutation and the plant number are shown in black. (b) and (c) Large fragment deletions in T0 transgenic plants caused by different target sites and a schematic diagram. The designations a, b, c, d and e refer to different types of deletions: ‘a’ indicates a 52‐bp deletion between target 3 and target 4; ‘b’ indicate a 152‐bp deletion between target 1 and target 4; ‘c’ and ‘e’ indicate a 100‐bp deletion between target 1 and target 3; ‘d’ indicates a 71‐bp deletion between target 1 and target 2 and a 52‐bp deletion between target 3 and target 4.

To identify the mutations induced by the CRISPR/Cas9 system, 114 single clones from 22 transgenic lines were sequenced. Of these, 74 (64.91%) had mutations in the T4 site and 60 (52.63%) in the T1 site (Figure 6a), while the mutation efficiency in the T2 and T3 sites was much lower, possibly due to the relatively high GC content in those sites (Table 1). In the T1 and T4 sites, short insertions (+1), short deletions (−1, −2, −4, −5, −6, −8, −10, −11) and large deletions (−52, −57) were found. The 1‐bp deletion was the most common mutation in the T1 site and appeared in seven independent lines (#39, #41,#42, #45, #47, #51 and #69), while the 1‐ to ~2‐bp deletion was detected for T4 in 12 independent lines (#37, #38, #41, #42, #47, #49, #50, #52, #54, #60, #61 and #68) (Figure 6a).

In addition, we observed that the large deletions were often induced by a combination of effects from the different targets. As shown in Figure 6b, four different large deletions were found in five transgenic lines (#38, #41, #47, #52 and #69). A 52‐bp deletion was detected in #38 and #52 between T3 and T4, a 100‐bp deletion in lines #47 and #69 between T1 and T3, a 71‐bp deletion in line #52 between T1 and T2 and a 152‐bp deletion in line #41 between T1 and T4 (Figure 6b,c). This indicated that the CRISPR/Cas9 system can be used for precise genome editing in the first generation.

### Characterization of mutations in mutant lines in the first generation

In order to further understand the mutation efficiency of various targets, we counted the mutagenesis of four targets sites in the transgenic T0 plant. The mutation efficiency in the sites T1 (27.78%), T3 (16.67%) and T4 (25.00%) were much higher than T2 (5.55%). Thirteen of 18 transgenic lines with mutation at target 4 were biallelic mutation and 6 of 20 for target 1 (Table 2). In addition, no biallelic mutation was found in T2 and T4 sites. This indicated that the target with high efficiency would improve the opportunity to get the biallelic mutation lines in the first generation.

**Table 2:** Summary of mutations about four targets in transgenic T0 plant of grape

Target	Transgenic line analysed	Transgenic line with mutation	% Mutation frequency	Monoallelic Mutant line	Biallelic mutant lines
1	72	20	27.78	14	6
2	72	4	5.55	4	0
3	72	12	16.67	12	0
4	72	18	25.00	5	13

For each target, the mutation frequency (%) was calculated based on the number of transgenic lines with mutation out of the analysed transgenic lines. Five to twenty‐four clones for independent mutant lines were sequenced to identify the mutations.

**Figure 7:**
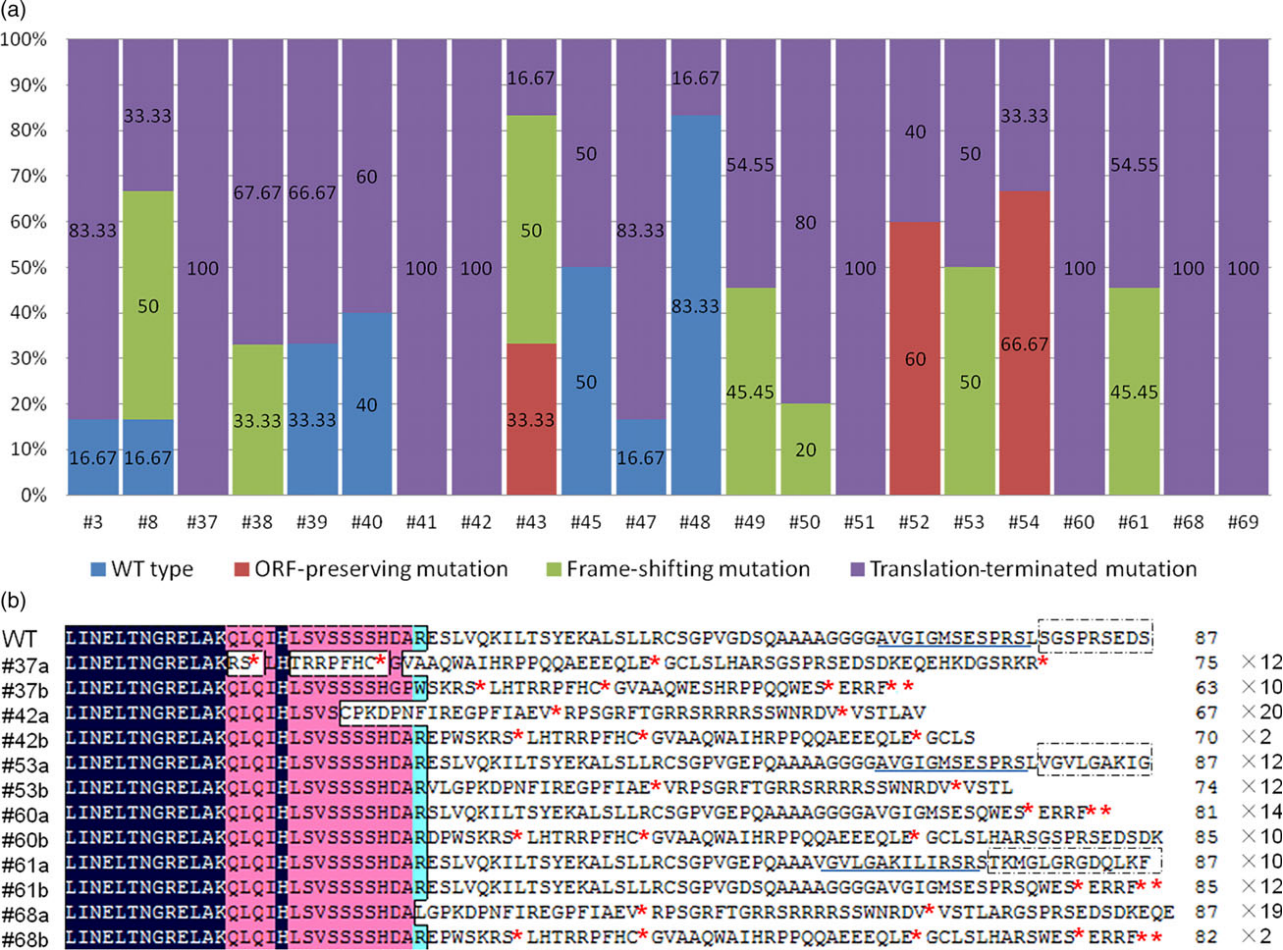
Summary of mutations in mutant transgenic T0 plants. (a) The frequency of different mutation types in dependent mutant lines. Three hundred clones from 22 independent transgenic lines were randomly selected for sequencing analysis. (b) The biallelic mutant lines in the first generation. ‘a’ and ‘b’ refer to the two alleles. ‘*’ indicates that translation is terminated. The sequence in the box refers to a transcoding mutation compared to wild type (WT). The numbers of clones detected with this mutation and the numbers of predicted amino acids are shown in black.

In order to identify the biallelic mutant lines, 5–24 randomly selected clones for every independent line were sequenced. Fifteen of 22 transgenic lines were found to contain biallelic mutations and seven were monoallelic mutation (Figure 4). However, three of 15 biallelic mutation lines were ORF‐preserving mutations and only 12 lines were loss of function in two alleles (Figure 7a). The deduced amino acid sequences from the mutated DNA sequences of six biallelic mutant lines (#37, #42, #53, #60, #61 and #68) are shown in Figure 7b. A mutation that would result in the early termination of translation of two different alleles was found in four selected lines (#37, #42, #60 and #68). However, in #53 and #61, this mutation was only found in one allele, and the other was a transcoding mutation within the region of the selected 87 amino acids (Figure 7). This indicated that biallelic mutant lines can be efficiently obtained using this CRISPR/Cas9 system in grape.

### Off‐target analysis

To detect off‐target events, the 12 transgenic lines with biallelic mutations and WT plants were selected for sequence analysis. The putative off‐target sites for each target were predicted with the online tool CRISPR‐P. Six putative off‐target sites, which may produce the mutations in the six different genes, were selected for further study (Table 3). The primers used for amplifying the off‐target areas are listed in Table S1. The PCR products were purified and sequenced using the forward primer, which had also been used for the amplification. No mutation was found in any of the 72 (12 lines × 6 putative off‐target sites) sequenced samples (Table 3).

**Table 3:** Off‐target analysis of the T0 plants

Target	Off‐target sites	Putative off‐target sequences	Putative off‐target genes and regions	Putative off‐target loci	Number of examined lines	Number of lines with off‐targets
1	1	AAATGATGCCCGTGTATCTT	VIT_19s0015g01740/exon	19:+10436739	12	0
	2	ACATGACGCCTTTGGAACCT	VIT_08s0040g00520/intro	8:+11459692	12	0
2	3	GCAGAGTTGTGGCTGCCCAG	VIT_00s0525g00040/exon	Un:+31238127	12	0
3	4	CCGGAGGCCTCCGCAGCAGA	VIT_04s0044g01470/exon	4:‐22994844	12	0
	5	CCACCAGCAGCGGCAGCAGG	VIT_02s0025g04670/exon	2:‐4232312	12	0
4	6	GAGACTCCATGCTTGCTCAT	VIT_13s0084g00600/exon	13:+19640196	12	0

Twelve biallelic mutant lines were used for the off‐target analysis. The off‐target sites of four targets were selected according to their putative off‐target efficiency and regions.

### Knockout *VvWRKY52* in Thompson Seedless enhances resistance to *Botrytis cinerea*

**Figure 8:**
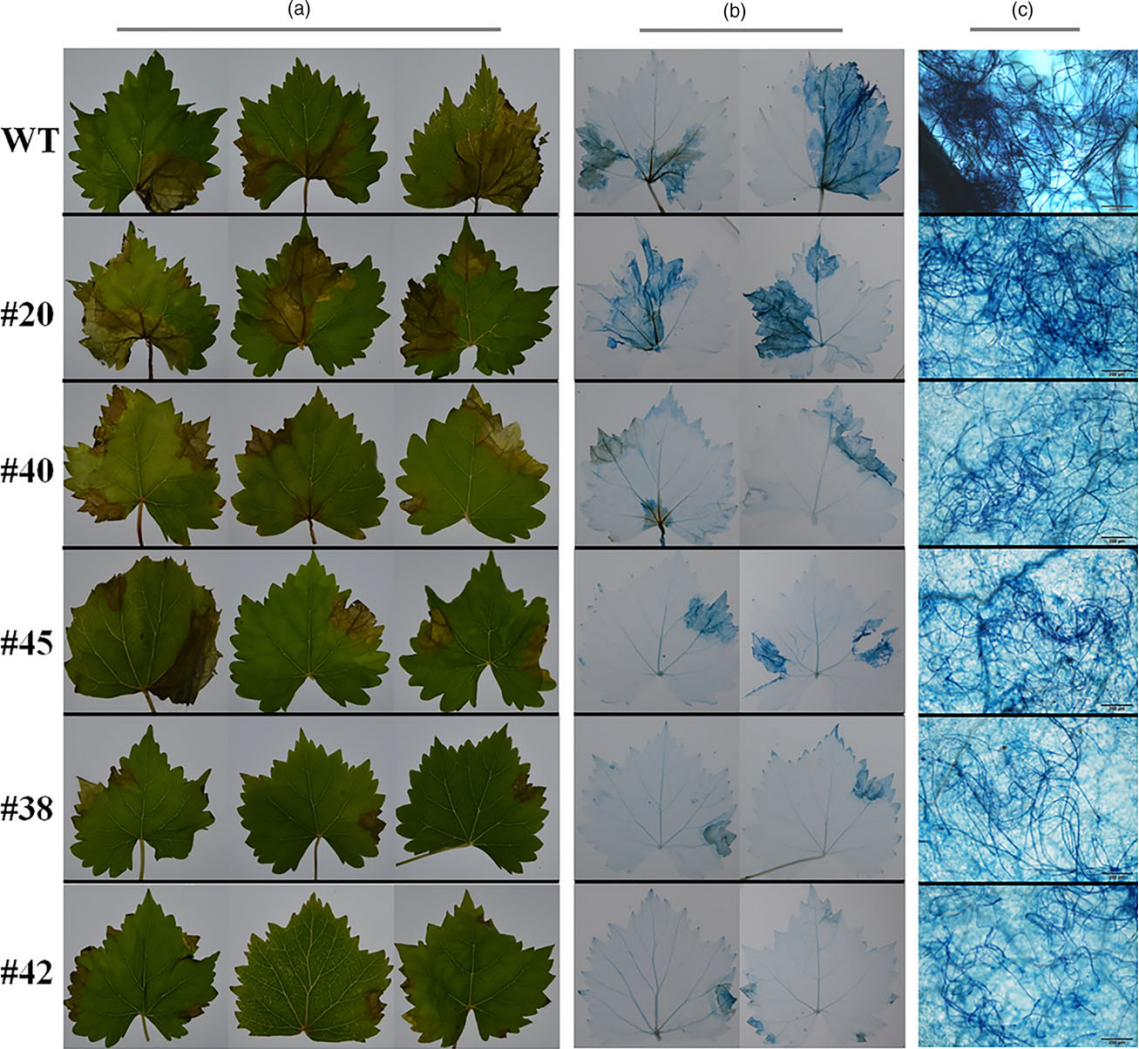
Phenotype of *VvWRKY52* transgenic lines inoculated with *Botrytis cinerea* at 5 days postinoculation. (a) Transgenic lines (#20, #40, #45, #38, #42) and wild‐type (WT) plants were infected with *B. cinerea*. Leaves were photographed 5 days postinoculation. Lines #20 had the T‐DNA insertion but no mutations in the *VvWRKY52* sequence. Lines #40 and #45 contained single allele mutations, and lines #38 and #42 contained biallelic mutations. (b) and (c) Plant cell death and fungal structures were stained with trypan blue at 5 days postinoculation. Scale bar = 200 μm.

**Figure 9:**
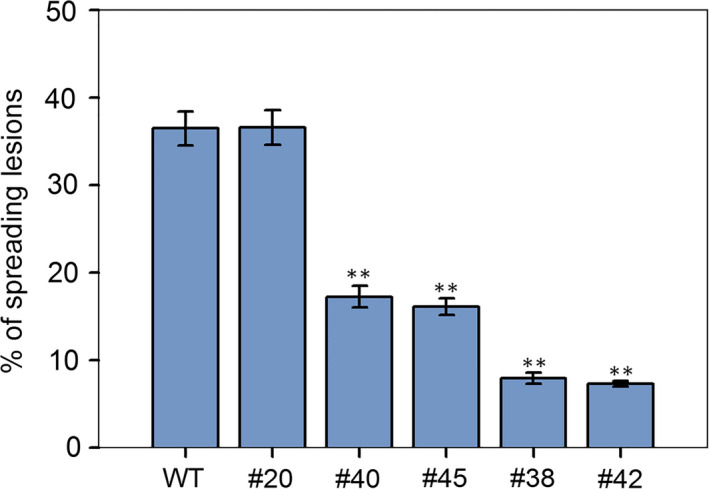
The percentage of spreading lesions counted at 5 days after inoculation. Lines #20 had the T‐DNA insertion, but no mutations. Lines #40 and #45 contained single allele mutations, and lines #38 and #42 contained biallelic mutations. The mean ± SD from three independent experiments is represented by bars. Asterisks indicate the statistical significance between transgenic lines and wild‐type (WT) plants (**P < 0.01, Student's *t‐*test).

To prove whether the targeted mutations of *VvWRKY52* affect the resistance of Thompson Seedless against *Botrytis cinerea*, the phenotypes of the WT and transgenic lines #20, #40, #45, #38 and #42 were selected for further analysis. We observed that in the detached leaves of four transgenic lines (#40, #45, #38 and #42) with mutant, the resistance to *B. cinerea* was found to be increased compared with WT plant at 5 days postinoculation. Interestingly, transgenic lines with biallelic mutant (#38 and #42) showed higher resistance than lines with monoallelic mutant (#40 and #45) and line without mutant (#20) showed no significant difference compared with WT plant (Figure 8a). We also observed the cell death and fungal structures of leaves from different phenotypes at 5 days postinoculation (Figure 8b,c). The largest cell death was found in WT plant and line #20, followed by lines #40 and #45, and lines #38 and #42 had the smallest (Figure 8b). The *B. cinerea* colonies growing on the WT plant and line #20 were the largest, followed by those on lines #40 and #45, while lines #38 and #42 had the smallest colonies (Figure 8c). These results are consistent with the phenotype in Figure 8a. In addition, we counted the percentage of spreading lesions on the phenotypes of the WT and transgenic lines at 5 days after inoculation (Figure 9). The results are consistent with disease phenotype in Figure 8a and the cell death in Figure 8b.

## Discussion

The use of the CRISPR/Cas9 system has developed rapidly in recent years (Ma *et al*., 2016), and the technology is being applied to gene functional analysis and molecular breeding (Cao *et al*., 2016; Jia *et al*., 2016; Ueta *et al*., 2017; Wang *et al*., 2014, 2016a; Yang, 2017). In grape, it has been shown to be effective with both protoplasts (Malnoy *et al*., 2016) and transgenic suspension cells (Ren *et al*., 2016), and appropriate target sites have been identified in the *V. vinifera* genome (Wang *et al*., 2016b). However, it has not been established whether this system could efficiently produce biallelic mutations, or the extent of off‐targeting in the first generation: the former issue is especially important for woody plants with long reproductive cycles (Fan *et al*., 2015).

Grape is one of the most economically important fruit trees grown worldwide, and the efficient production of biallelic mutations in the first generation would be highly significant. In recent years, many CRISPR/Cas9 toolkits for multiplex genome editing in plants have been reported (Ma and Liu, 2016; Xing *et al*., 2014), making it possible to design multiple targets for one gene and significantly improve the mutation efficiency, accelerating the application of this technique. In this current study, we designed four targets for one gene (Figure 1a) and the expression cassettes were inserted into a binary vector (Figure 1b), which had previously been shown to work in poplar (Fan *et al*., 2015).

Organogenesis and somatic embryogenesis are two commonly used strategies for grape transformation (Martinelli *et al*., 1993; Robacker, 1993). Compared to the organogenesis pathway, somatic embryogenesis has a very low chimera rate and is the more frequently used method for regeneration in grape (Gambino *et al*., 2007), so we used this approach, following a protocol described in Dhekney *et al*. (2012), with minor changes. An overview is provided in Figures 2 and 3. We used the PEM for transformation instead of the SE, which has been previously reported (Zhou *et al*., 2014b), and which we found to be effective.

Previous studies have shown that the mutation efficiency of CRISPR/Cas9 varies widely (Ma *et al*., 2016), and it can be affected by factors such as plant species, target sequence, Cas9 promoter and sgRNA sequences and transformation method (Ma *et al*., 2016). In our study, we obtained 72 regeneration lines through Agrobacterium‐mediated transformation, all of which had the T‐DNA insertion (Figure S1), indicating that the method described here is optimal. Twenty‐two (31%) of the 72 transgenic lines contained mutations in the target sites, which is a high mutation rate for grape. CRISPR/Cas9 is known to cause biallelic mutations in several plant species, such as rice (Zhang *et al*., 2014), *A. thaliana* (Wang *et al*., 2015) and maize (Svitashev *et al*., 2015). Moreover, in our study, 15 of the 22 transgenic lines with mutations were shown to be biallelic (Figures 4 and 7), making this an attractive method to use in grape. We observed no difference in phenotype between the different genotypes (Figure 5), indicating that knocking out this gene did not influence the growth and development of the plants. WRKY transcription factor family has been shown to play roles in biotic stress responses (Guo *et al*., 2014). To identify the function of *VvWRKY52* in biotic stress, we compared the resistance of the phenotypes of WT and five transgenic lines against *B. cinerea*. We found that knocking out *VvWRKY52* in grape increased the resistance to *B. cinerea* (Figures 8 and 9). It indicated that the CRISPR/Cas9 system represents a useful tool for gene functional analysis in grape.

The CRISPR/Cas9 system generates insertion or deletion mutations; for example, a study in apple revealed that all the mutations were short insertions or short deletions (Nishitani *et al*., 2016). Here, we found that most mutations were short deletions (Figure 6a), while a previous study in grape showed that most of the mutations were short insertions (Ren *et al*., 2016). We speculate that this difference reflects the use of different genotypes (Chardonnay/Thompson Seedless) or different sample types (suspension cells/T0 plants). It has been reported that multiple close targets in one gene in woody plants can result in large deletions (Fan *et al*., 2015) and, indeed, we identified four different large deletions (Figure 6b,c), which were induced by cutting at two or more target sites. This indicated that the CRISPR/Cas9 system can be used for precise genome editing in the first generation.

We concluded that the CRISPR/Cas9 system can be used to efficiently generate biallelic mutant lines in the first generation. However, as off‐target mutation events may influence the application of this system (Ren *et al*., 2016), we also investigated their occurrence. A previous study showed that a GC content of 50%–70% in the target sequences usually results in a high editing efficiency (Ma *et al*., 2015), which is consistent with our results. The GC contents of the four selected targets are listed in Table 1. The mutation efficiency was much higher in the T1 and T4 sites, which had GC contents of 55% and 65%, respectively. Targets with high editing efficiency may also be associated with a greater risk of off‐targeting, which we hypothesize might be the case with the T1 and T4 sites. We selected six potential off‐target sites for further analysis (Table 3) and screened all 12 transgenic lines with biallelic mutations, as well as WT plants. No mutation was found in any of the 72 (12 lines × 6 putative off‐target sites) sequenced samples (Table 3), suggesting a very low number of off‐target events in grape. To summarize, our study demonstrated that the CRISPR/Cas9 system is a useful tool for efficiently generating biallelic mutation lines in the first generation of grape transformants, and will likely accelerate grape gene functional research and molecular breeding.

## Materials and methods

### Plant material and cultures

Thompson Seedless seedlings were grown in the grape germplasm resources orchard at the Northwest A & F University, Yangling, Shaanxi, China. The embryogenic callus was induced from floral explants of Thompson Seedless according to a previously published protocol (Gribaudo *et al*., 2004). The embryogenic calli were transferred to X6 medium (Caisson, MSP24‐1LT) to form PEM, which were used for grape transformation. All cultures described above were maintained in the dark at 26 °C.

### Vector construction

Genomic DNA was extracted from T0 transformed and WT plants with the plant genomic DNA extraction kit (Bioteke, Beijing, China), according to the user manual. *VvWRKY52* was amplified from Thompson Seedless genomic DNA using the PrimeSTAR^®^ Max DNA Polymerase kit (Takara, Dalian, China) with gene‐specific primers (*VvWRKY52*‐Target‐F: 5′‐ATGGAGAACATGGGAAGTTGGG‐3′; *VvWRKY52*‐Target‐R: 5′‐TTGAATCATATGAACGGATGGATG‐3′), designed based on the homologous gene GSVIVT01028718001 or VIT_16s0050g02510 from the Grape Genome Sequence (http://www.genoscope.cns.fr) or EnsemblPlants (http://plants.ensembl.org/index.html), respectively. The PCR product was cloned into the pClone007 Simple Vector (TSINGKE), and the sequence was then used to design CRISPR/Cas9 target sites, with the online tools CRISPR‐P (http://cbi.hzau.edu.cn/crispr/) and CRISPR RGEN (http://www.rgenome.net/). Four targets were selected based on their GC content, location in the gene and off‐target situation.

The binary vector pYLCRISPR/Cas9P_35S_‐N and four helper plasmids (PYLsgRNA‐*LacZ*‐AtU3d, ‐AtU3b, ‐AtU6‐1, ‐AtU6‐29) (Ma *et al*., 2015) were used to generate the CRISPR/Cas9 construct, which included four sgRNA cassettes, following the multiple sgRNA Golden Gate Cloning assembly protocol (Ma and Liu, 2016). The primers used in this experiment are listed in Table S1.

### Plant transformation

The PEM, maintained in the dark at 26 °C, was transferred to fresh X6 medium for 1 week and then used for grape transformation. The binary vector was introduced into *A. tumefaciens* strain EHA105 using the freeze–thaw method (Wise *et al*., 2006). The *A. tumefaciens* culture used for co‐cultivation was prepared as previously described (Zhou *et al*., 2014b). Agrobacterium‐mediated transformation of the PEM was performed according to Dhekney *et al*. (2012) with minor modifications. Briefly, the bacterial culture (OD600, 0.4–0.6) was incubated with the PEM for 7 min, and the PEM was then transferred onto filter paper to remove excess bacteria. Blotted PEM was transferred to a Petri dish containing two layers of filter paper with liquid DM medium (DKW basal salts, 2.0 mg/L each of thiamine‐HCl and glycine, 1.0 mg/L nicotinic acid, 0.3 g/L KNO 1.0 g/L myo‐inositol, 30 g/L sucrose, 5.0 mm 6‐benzyladenine, 2.5 mm 2‐naphthoxyacetic acid and 2.5 mm 2,4‐dichlorophenoxyacetic acid, pH 5.7) and co‐cultivated in the dark at 26 °C for 3 days. After 3 days, the PEM was transferred to solid DM medium containing 200 mg/L carbenicillin, 200 mg/L cefotaxime and 75 mg/L kanamycin, for 1 month in the dark at 26 °C. The resulting callus was transferred to X6 medium with 200 mg/L carbenicillin, 200 mg/L cefotaxime and 75 mg/L kanamycin. Petri dishes were placed in the dark for the development of transgenic SE lines. SE at the late cotyledonous stage was transferred onto MS1B medium (MS salts and vitamins, 0.1 g/L myo‐inositol, 20.0 g/L sucrose, 1.0 mm 6‐benzyladenine, and 7.0 g/L TC agar, pH 5.8) under a 16‐h photoperiod with white fluorescent lights, to regenerate the plants.

### Detection of mutations

To identify stable transgenic lines, vector‐specific primers (*NPTII* ‐F: 5′‐AGAGGCTATTCGGCTATGACTG‐3′; *NPTII* ‐R: 5′‐CAAGCTCTTCAGCAATATCACG‐3′) were used. The potential edited area of *VvWRKY52* was amplified using gene‐specific primers (*VvWRKY52*‐Target‐F; *VvWRKY52*‐Target‐R) with the PrimeSTAR^®^ Max DNA Polymerase kit (Takara) from stable transgenic lines and WT plants. The PCR product was purified and sequenced using the specific primer, *VvWRKY52*‐Target‐F. All mutant transgenic lines were selected for the next identification step. PCR products from transgenic lines whose sequence showed a bimodal pattern were inserted into the pClone007 Simple Vector (TSINGKE). Single clones were sequenced and 5–20 single clones from each stable transgenic line with mutants were selected for further analysis to identify the mutation. DNAMAN (version 4.0; Lynnon Biosoft, Inc., San Ramon, CA) was used for alignment analysis.

### Off‐target analysis

The stable transgenic lines with biallelic mutants were used for off‐target analysis. The potential off‐target sites of the four targets, predicted using the online CRISPR‐P (http://cbi.hzau.edu.cn/crispr/) tool, were selected for further analysis (Table 3). Specific primers were designed and used to amplify the genomic DNA fragments with potential off‐target sites (Table S2), and then, the genomic DNA fragments were sequenced.

### Inoculation of grape with pathogen

*Botrytis cinerea* isolated from grape was maintained on potato glucose agar medium in the dark at 25 °C. After 21 days, conidia were used for inoculation. The *B. cinerea* conidial suspension (1.5 × 10^6^ conidia/mL) was prepared and used for inoculation by spraying as previously described (Wang *et al*., 2017). Detached leaves with the same size were selected and transferred to a bed of 0.8% agar in trays quickly. The leaves were sprayed with conidial suspension. Then, preservative film was used to cover the trays to ensure a relative humidity of 90%–100% as previously described (Wan *et al*., 2015). Spraying with distilled water was used as the control. All trays were maintained in the dark for 24 h, then in a light/dark (16/8‐h) regime at 22 °C. For each different lines and WT plant, at least 18 leaves from three biological replicates were tested. The percentage of spreading lesions was counted at 5 days after inoculation (Wan *et al*., 2015). Cell death and the fungal structures were stained with trypan blue as previously described (Wang *et al*., 2017).

### Statistical analysis

Microsoft Excel (Microsoft Corporation, Redmond, WA) and Sigma plot (v. 10.0; Systat Inc., Point Richmond, CA) were used for data analysis. SPSS Statistics 17.0 software (IBM China Company Ltd., Beijing, China) was used to assess the significant differences through paired *t*‐tests. All experiments were repeated three times as independent analyses.

## Author contributions

X. Wang and X.H. Wang designed the study. X.H. Wang and M. Tu contributed to the experiments. X.H. Wang and D. Wang constructed the vectors. M. Tu, D. Wang and Y. Li performed data analysis. Z. Li, J. Liu and Y. Wang assisted with the data analysis. X.H. Wang and X. Wang wrote the manuscript. All of the authors approved the final manuscript.

## Conflict of interest

The authors declare no conflicts of interest.

## Supporting information


**Figure S1** Schematic map of *VvWRKY52* location and two alleles of *VvWRKY52* in Thompson Seedless. Allele I and Allele II are part of the coding sequences of *VvWRKY52*. ‘*’ and red line indicated the difference.

**Figure S2** Identification of T‐DNA insertion of 72 transgenic lines. ‘P’ means positive control and WT (wild type) was negative control.

**Figure S3** The DNA fragments from independent transgenic lines were amplified for sequencing. The number indicated different transgenic lines. WT indicated non‐transgenic line.

**Table S1** Primers used for off‐target analysis.
**Table S2** Primers used for vector construction.
**Table S3** Mutant information at target 1.
**Table S4** Mutant information at target 4.
