# Supplementary figures and images for: CRISPR/Cas9‐mediated efficient targeted mutagenesis in grape in the first generation
Source: Plant Biotechnol J. 2017 Nov 10;16(4):844–55. doi: 10.1111/pbi.12832 (PMC5866948; doi:10.1111/pbi.12832)

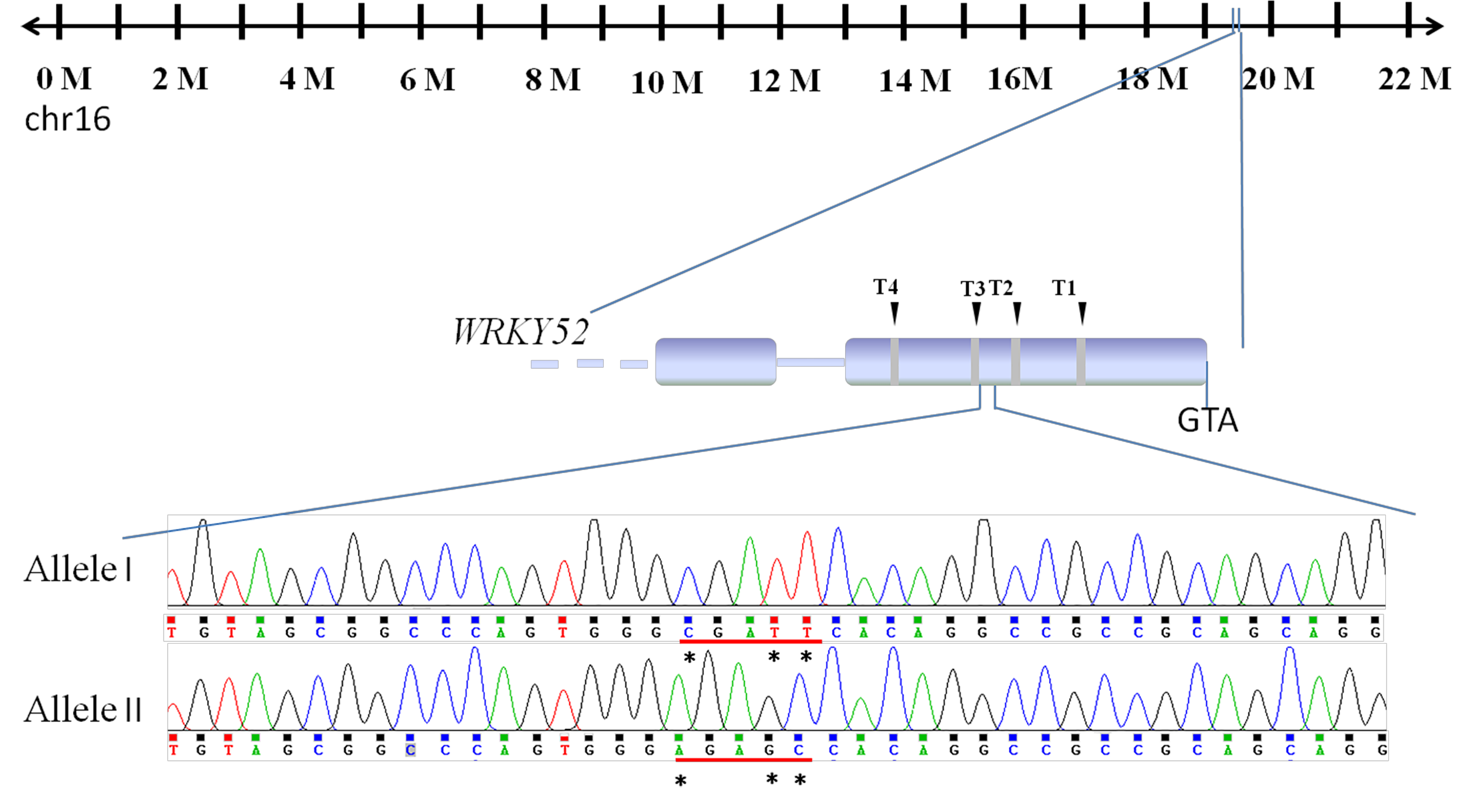

Supplement: Supplementary file 1 — Figure S1 Schematic map of VvWRKY52 location and two alleles of VvWRKY52 in Thompson Seedless. Allele I and Allele II are part of the coding sequences of VvWRKY52. ‘*’ and red line indicated the difference. [file PBI-16-844-s004.tif]

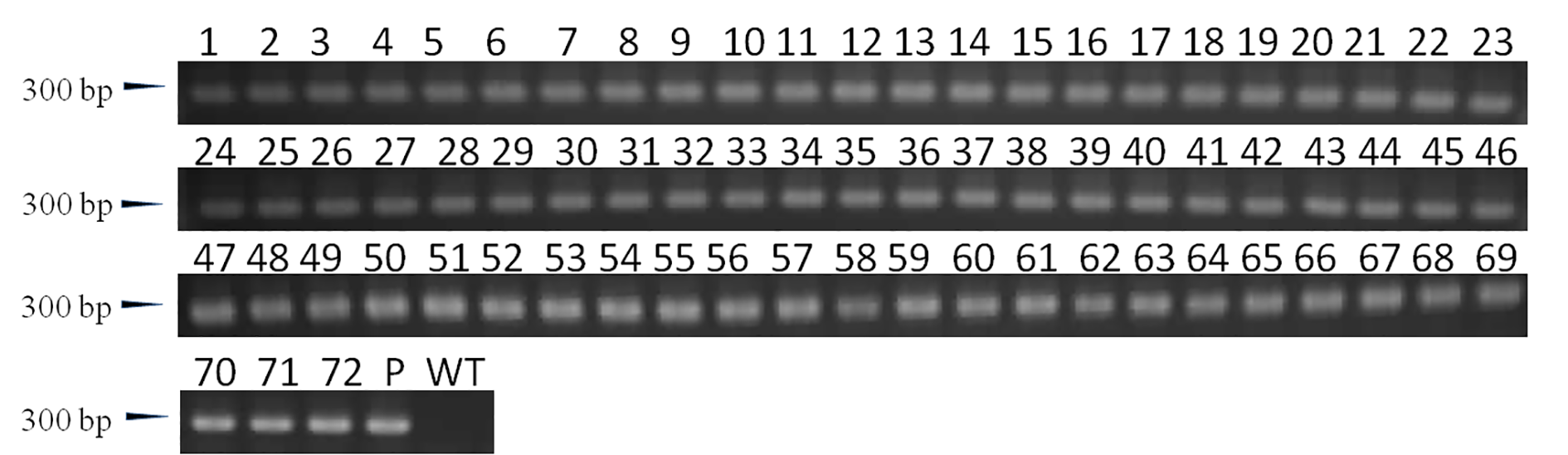

Supplement: Supplementary file 2 — Figure S2 Identification of T‐DNA insertion of 72 transgenic lines. ‘P’ means positive control and WT (wild type) was negative control. [file PBI-16-844-s003.tif]

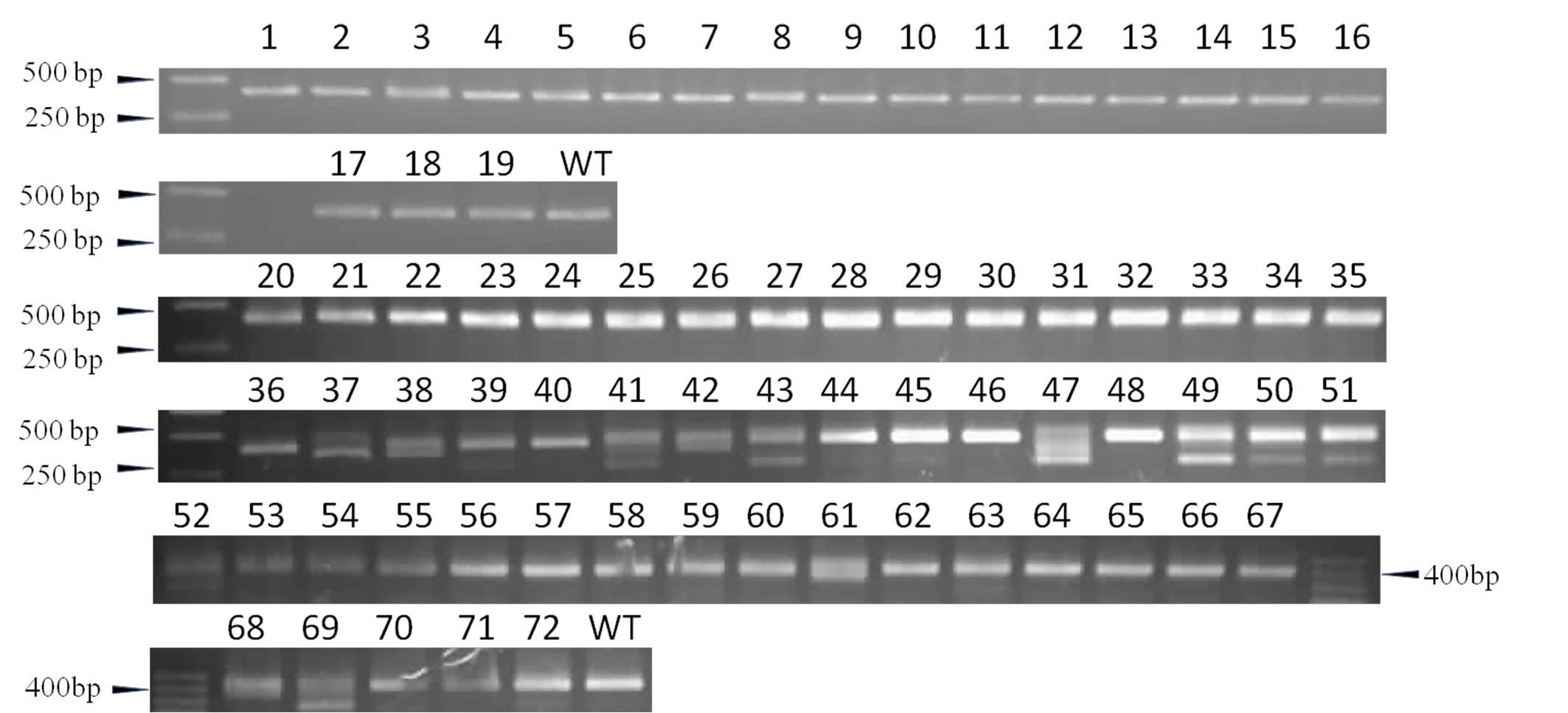

Supplement: Supplementary file 3 — Figure S3 The DNA fragments from independent transgenic lines were amplified for sequencing. The number indicated different transgenic lines. WT indicated non‐transgenic line. [file PBI-16-844-s002.tif]
