# Supplementary material for: CRISPR/Cas9‐mediated efficient targeted mutagenesis in grape in the first generation
Source: Plant Biotechnol J. 2017 Nov 10;16(4):844–55. doi: 10.1111/pbi.12832 (PMC5866948; doi:10.1111/pbi.12832)
Supplement: Supplementary file 4 — Table S1 Primers used for off‐target analysis. Table S2 Primers used for vector construction. Table S3 Mutant information at target 1. Table S4 Mutant information at target 4. [file PBI-16-844-s001.docx]

Table S1. Primers used for vector construction

| Primer name | Primer sequence |
| --- | --- |
| gRT1+ | acatgacgcccgtgaatcctgttttagagctagaaat-3’ |
| gRT2+ | gctgaggtgtagcggcccaggttttagagctagaaat-3 |
| gRT3+ | cacaggccgccgcagcagggttttagagctagaaat-3 |
| gRT4+ | agtctccacgctcgctcagtgttttagagctagaaat-3’ |
| AtU3bT1- | aggattcacgggcgtcatg**T**gaccaatgttgctcc-3’ |
| AtU6-1T2- | ctgggccgctacacctcag**C**aatcactacttcgtct-3’ |
| AtU6-29T3- | cctgctgcggcggcctgtg**C**aatctcttagtcgact-3’ |
| AtU3dT4- | actgagcgagcgtggagac**T**gaccaatggtgctttg-3’ |

Table S2. Primers used for off-target analysis

| Off-target site | Forwards primer | Reverse primer |
| --- | --- | --- |
| 1 | CTTAAATAGAGAAAGACTGCAGGGAC | CCAAGCTAGGCTTTTGTCAAGGCATA |
| 2 | CTCTCCAAATTCTACCTAAGATTTCTTTA | CAAATCTTGAAATTGGGGCTTT |
| 3 | TTGAACCAGACATTGTGGTTTATAACA | GTTGTATCATATTATCCAAAAGCTCATAAC |
| 4 | ATATGCTGGCCTCTTCAGATTTTG | ACTGTATTTTTGCTATCTGTCTCTCTTTC |
| 5 | TTTGAAGGCTTTTGTTCATGGG | GGAAGCAACATGTACAGTTCTCAATG |
| 6 | GTAGTCCTCCGATCTCTCTCACTGC | CTAATTCTCTTAGCGGTTGTCATGC |

Table S3. Mutant information at target 1

| Mutant lines | Number of mutant type at allele 1 | WT type at allele 1 | Number of mutant type at allele II | WT type at allele II |
| --- | --- | --- | --- | --- |
| #37 | 1 | No | 1 | No |
| #38 | 0 | Yes | 1 | No |
| #39 | 1 | No | 0 | Yes |
| #42 | 1 | No | 1 | No |
| #45 | 1 | No | 0 | Yes |
| #51 | 1 | No | 0 | Yes |
| #60 | 1 | No | 1 | No |
| #68 | 1 | No | 1 | No |
| #69 | 1 | No | 1 | No |

Table S4. Mutant information at target 4

| Mutant lines | Number of mutant type at allele 1 | WT type at allele 1 | Number of mutant type at allele II | WT type at allele II |
| --- | --- | --- | --- | --- |
| #37 | 1 | No | 1 | No |
| #38 | 1 | No | 1 | No |
| #42 | 1 | No | 1 | No |
| #51 | 0 | Yes | 1 | No |
| #60 | 1 | No | 1 | No |
| #61 | 1 | No | 1 | No |
| #69 | 1 | No | 1 | No |
